# Vertical and Orovestibular Forces Generated by Beta-Titanium and Stainless-Steel Rectangular Wires in Labial and Fully Customized Lingual Bracket Systems

**DOI:** 10.3390/ma14195632

**Published:** 2021-09-28

**Authors:** Chrystalla Kyprianou, Athina Chatzigianni, Nikolaos Daratsianos, Christoph Bourauel

**Affiliations:** 1 Department of Oral Medical Technology, School of Dentistry, Faculty of Medicine, University of Bonn, 53111 Bonn, Germany; bourauel@uni-bonn.de; 2 Department of Orthodontics, School of Health Sciences, Faculty of Dentistry, Aristotle University of Thessaloniki, 54124 Thessaloniki, Greece; achatzigianni@dent.auth.gr; 3 Department of Orthodontics, School of Dentistry, Faculty of Medicine, University of Bonn, 53111 Bonn, Germany; nikolaos.daratsianos@uni-bonn.de

**Keywords:** orthodontics, lingual brackets, labial brackets, wires, forces, brackets, rectangular wires, stainless steel, beta titanium, TMA wires

## Abstract

This study aimed to investigate the force values exerted from rectangular wires when combined with conventional labial and fully customized lingual appliances under predefined, idealized activation. Fully customized lingual brackets of two brands Incognito™ (3M Unitek, Monrovia, CA, USA) and WIN (DW Lingual Systems, Bad Essen, Germany) and labial brackets of another brand, discovery^®^ MIM and discovery^®^ smart systems (Dentaurum, Ispringen, Germany), were chosen. Stainless-steel and beta-titanium wires of 0.018” × 0.025” were examined. For Incognito^TM^, 0.0182” × 0.025” beta-titanium wires were tested. Intrusion/extrusion and orovestibular movements were performed in a range of 0.2 mm, and the forces were recorded for each 0.1 mm of the movement. Mean values and standard deviations were calculated for all measurements, and ANOVA was performed for statistical analysis. Slight differences were observed between the forces generated from beta-titanium and stainless-steel wires. The same wire generated in some cases 5–53% higher forces with the lingual appliance due to the vertical orientation of the long walls during intrusion/extrusion and increased wire stiffness at the anterior region. Beta-titanium and stainless-steel 0.018” × 0.025” wires can generate similar force values during the final stages of the orthodontic therapy; thus, possibly only one of the two alloys could be used in each orthodontic wire sequence.

## 1. Introduction

Throughout the years, fixed appliances have evolved in an effort to ensure treatment efficacy, comfort and aesthetics, as well as reduced chair time and treatment duration. The superiority of modern treatment protocols is the topic of choice for many researchers, while others look into the possible side effects of fixed appliances, such as white spot lesions, enamel damage after debonding, root resorption or changes in the pulp metabolic activity during orthodontic therapy [1,2,3,4]. The introduction of new materials and the continuous improvement of existing ones highlight the necessity for further research.

Conventional metallic bracket systems are now competing with self-ligating or esthetic labial orthodontic appliances, while lingual bracket systems have been gaining ground in the preference of both patients and orthodontic professionals during the last two decades. Kurz et al. (1982) presented the first lingual bracket system, and Wiechman et al. (2002) pioneered the fully customized lingual appliances [5,6]. In the years ahead, various researchers investigated the treatment effects and mechanical properties of these appliances. Slot morphology and dimensions, as well as the slot play of the lingual appliances, were repeatedly studied [7,8]. The forces generated from the fully customized lingual appliances were previously investigated, however, the existing results result in controversy [9,10,11].

At the same time, several biomaterials are being used for the production of orthodontic wires of various diameters or cross sections, and miscellaneous wire sequence protocols have been proposed by different scientists [12]. Titanium molybdenum alloy (TMA) was introduced in the 1980s as an intermediate between nickel titanium (NiTi) and stainless-steel (SS) wires with an elastic modulus of 10.5 Msi (72.4 GPa) [13]. Morinaga et al. (1988) developed a method for the design of titanium alloys, which allowed reduction in the Young’s modulus of these archwires [14]. Thermal treatments, which have the same effect on titanium wires, have been proposed by other scientists [15,16]. Kusy et al. (1983) calculated the ratios of the major properties, i.e., stiffness, strength and range of numerous alloys, including TMA and SS. According to these ratios, beta-titanium (β-Ti) shows lower strength, higher range and stiffness of one-third of SS [17]. The unique biomechanical properties of beta-titanium wires were investigated by several authors [18,19,20,21,22]. The TMA is still considered the most recent entrance in the production industry of conventional orthodontic wires, although new titanium alloys are proposed occasionally [23].

Regarding the comparison of different bracket/rectangular wire combinations, Daratsianos et al. (2016) and Tran et al. (2021) compared the torque capabilities of SS and β-Ti rectangular wires combined with labial and/or lingual appliances [8,24]. These investigators were driven by the fact that rectangular wires of the specific alloys are indicated as finishing wires when effective torque control is needed. In addition, particular archwires are ideal for segment stabilization and as a substitute of nickel titanium wires in cases of nickel allergies [25,26,27]. 

Based on the current literature, this study aimed to resolve the controversy over force values generated from fully customized lingual appliances and to compare the forces generated from stainless-steel and beta-titanium wires when combined with the selected bracket appliances. Furthermore, the authors intended to document the forces produced by rectangular wires of these alloys when combined with multiple bracket systems for the certain indications, which is something that has not been analyzed before. The experiment was performed under clearly predefined and idealized activation conditions. The null hypothesis was that there are no differences in the produced force levels of the tested bracket appliances and wire alloys.

## 2. Materials and Methods

### 2.1. Bracket Appliances and Wires

Four bracket appliances with 0.18 inch slots (Incognito™ lingual brackets (3M Unitek, Monrovia, CA, USA), WiN lingual brackets (DW Lingual Systems, Bad Essen, Germany), discovery^®^ MIM and discovery^®^ smart appliances (Dentaurum, Ispringen, Germany)) were combined with 0.018” × 0.025” β-Ti and 0.018” × 0.025” SS archwires. Specifically, Incognito^TM^ offers 0.0182” × 0.025” beta-titanium wires. Preformed archwires (Rematitan^®^ Special ideal arches/Remanium^®^ ideal arches (Dentaurum, Ispringen, Germany)) were used for the labial appliances. For the fully individualized bracket systems, the licensed laboratories constructed customized wires (i.e., mushroom shaped with straight lateral segments) in order to fit a moderately crowded mandibular arch with rotated premolars [28]. 

The physical, mechanical and thermal properties of the selected archwires are shown in Table 1. All wire dimensions and slot lengths (according to ISO27020:2019) were registered by using a manual ratchet thimble micrometer (Mitutoyo America Corporation, Aurora, IL, USA) and a digimatic caliper (Mitutoyo Digimatic 500-120, Mitutoyo Deutschland GmbH, Neuss, North Rhine-Westphalia, Germany).

### 2.2. Model

A full set of lingual brackets, archwires, transfer trays and setup models was received from each laboratory. For this investigation, only the setup models and the final customized wires were used. Prior to the customization of the Incognito^TM^ appliances, the respective laboratory received a scanned copy of the WiN setup model in order to reproduce the aligned arch and manufacture archwires with a similar therapeutic goal.

Resin replicas (Technovit^®^ 4004, Kulzer GmbH, Hanau, Germany) identical to the setup model received from the WiN laboratory were constructed. Forces were registered at three tooth positions: the canine (#33), the lateral incisor (#42) and the second premolar (#45). These teeth were removed from the casts in order to create space for the force/moment sensor. For both the labial and lingual appliances, a standard bonding procedure was followed. Wire splints were used to achieve slot leveling during the bonding procedure. The Transbond™ XT lightcure adhesive primer and paste (3M Unitek, Monrovia, CA, USA) and the Maximum Cure^®^ sealants A and B (Reliance Orthodontics, Itasca, IL, USA) were used for the labial and lingual appliances, respectively.

Interbracket distances were measured with the use of an electronic caliper (Mitutoyo Digimatic 500–120, Mitutoyo Deutschland GmbH, Neuss, Germany) on the resin casts and on photos of the bonded setup models. 

### 2.3. Apparatus

The experiment was performed in a temperature-controlled chamber (VEM 03/400, Heraeus Voetsch, Germany) of the orthodontic measurement and simulation system (OMSS) [29,30]. The chamber includes two force/moment sensors connected with three-dimensional positioning tables attached to stepping motors, which allow movement in the three planes of space. The apparatus was constructed based on the idea of a two-tooth model as described by Burstone and Koenig [31] and may be regarded as an electronic typodont, which continuously registers the force and moment values. 

### 2.4. Activation Procedure

**Figure 1:**
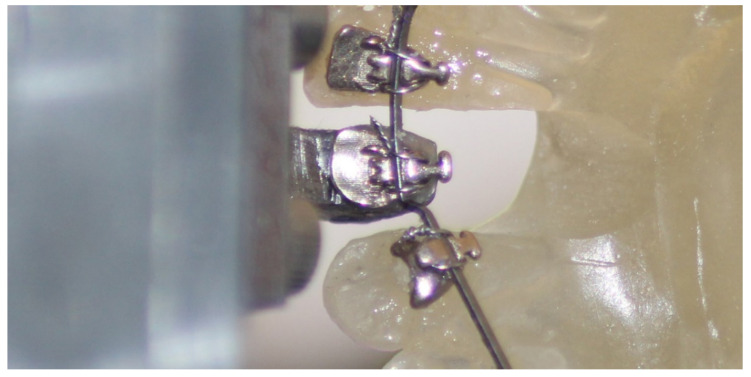
Resin cast bonded with the Incognito^TM^ appliance and adjusted in the OMSS chamber.

Each model was mounted in the OMSS chamber, and the sensor was adjusted in a position where the initial forces were neutralized. The selected wire piece was then ligated on the sensor and the neighboring teeth (Figure 1). The wires were ligated with Remanium^®^ short, preformed ligatures (Dentaurum, Ispringen, Germany). For the specific study, each bracket/wire combination was actively moved (successive steps of 0.02 mm) on the x-axis and z-axis, while the 3D sensors detected the generated forces. In the specific configuration, displacement on the x-axis represented intrusion/extrusion, and displacement on the z-axis represented orovestibular movement. Forces were registered at 0.1 mm, 0.2 mm and then backwards, so both positive and negative activation movements were applied on all systems. Each time a new model was tested, the system was neutralized in order to guarantee that forces were eliminated at the initial position. 

The activation process was performed separately at each tooth area, and each activation circle was repeated five times. The Incognito model was activated with new wires and new ligatures in each activation circle, while one set of wires was available for the WiN model; thus, the specific wire pieces were readjusted and religated for every repetition.

### 2.5. Statistical Analysis

For each group of five repetitions, the mean value and the standard deviation were calculated. Subsequently, all mean values were tested for normal distribution using the Kolmogorov–Smirnov test. The one-way analysis of variance (ANOVA) was performed in order to identify differences between the various bracket-wire combinations for each direction and each 0.1 mm of the activation procedure. Student–Newman–Keuls tests were chosen as post-hoc tests mainly to avoid Type 1 error. Student’s t-tests for equality of means were used for group comparisons between the two lingual and the two labial appliances, and the Levene’s test of equality of variances was used to compare standard deviations. The statistical analysis was performed with the SPSS Statistics software version 9 (IBM, Armonk, New York, NY, USA).

## 3. Results

Table 2 shows the measured slot specifications for all bracket types and the interbracket distance. Table 3 presents the measured wire dimensions.

In general, the generated forces ranged between 0.4 N and 4.1 N for all the bracket types combined with the β-Ti wires and between 0.6 N and 4.7 N for all bracket types combined with SS wires. 

### 3.1. Stainless-Steel Wires

Table 4 and Table 5 present the force mean values observed during the activation procedure of the SS wire. According to ANOVA results, statistically significant differences between the four appliances were observed in all cases apart from the oral movement of the lateral incisor and the premolar (Table 5; (#42; 0.1 mm; Fz; *p* = 0.699); (#42; 0.2 mm; Fz; *p* = 0.451); (#45; 0.2 mm; Fz; *p* = 0.388)). In addition, lingual appliances generated higher forces at the premolar area during intrusion and extrusion movement ((Table 4; #45; Fx; 0.1 mm; *p* = 0.000 and 0.2 mm; *p* = 0.000), (Table 5; #45; Fx; 0.1 mm; *p* = 0.000 and 0.2 mm; *p* = 0.022)). At the canine and lateral incisor area, lingual appliances generated higher forces only during extrusion movement (Table 5; (#33; Fx; 0.1 mm; *p* = 0.000 and 0.2 mm; *p* = 0.000), (#42; Fx; 0.1 mm; *p* = 0.000 and 0.2 mm; *p* = 0.000)). Group comparisons showed statistically significant differences between appliances of the same type, i.e., between the two labial appliances and between the two lingual systems.

### 3.2. Beta-Titanium Wires

Table 6 and Table 7 show the mean values recorded during the activation of the β-Ti wire. In agreement with the activation of the SS wire, ANOVA proved that the forces generated from the lingual systems were significantly higher than those generated by the labial appliances during the intrusion and the extrusion of the premolar ((Table 6; #45; Fx; 0.1 mm; *p* = 0.000 and 0.2 mm; *p* = 0.000), (Table 7; #45; Fx; 0.1 mm; *p* = 0.000 and 0.2 mm; *p* = 0.000)). The lateral incisor presented the same pattern ((Table 6; #42; Fx; 0.1 mm; *p* = 0.000), (Table 7; #42; Fx; 0.1 mm; *p* = 0.000 and 0.2 mm; *p* = 0.000)). At the canine area, forces produced by labial appliances were lower during extrusion (Table 7; #33; Fx; 0.1 mm; *p* = 0.000 and 0.2 mm; *p* = 0.000) and orovestibular activation ((Table 6; #33; Fz; 0.1 mm; *p* = 0.000 and 0.2 mm; *p* = 0.000), (Table 7; #33; Fz; 0.1 mm; *p* = 0.000 and 0.2 mm; *p* = 0.000)). During the orovestibular movement of the lateral incisor, lingual and labial appliances generated approximately the same force values ((Table 6; #42; Fz; 0.1 mm; *p* = 0.264), (Table 7; #42; Fz; 0.1 mm; *p* = 0.054)). Student’s t-tests showed several differences between the brackets of the same type.

**Figure 2:**
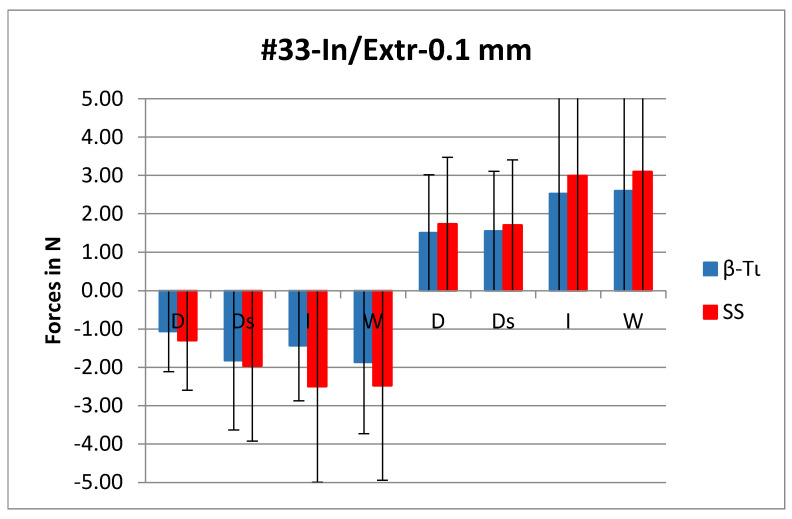
Graphic representation of the forces generated at 0.1 mm of the intrusion and extrusion of the canine (#33). SS: stainless steel; β-Ti: beta titanium; D: discovery^®^ MIM; Ds: discovery^®^ smart; W: WiN; I: Incognito™; N: Newton.

**Figure 3:**
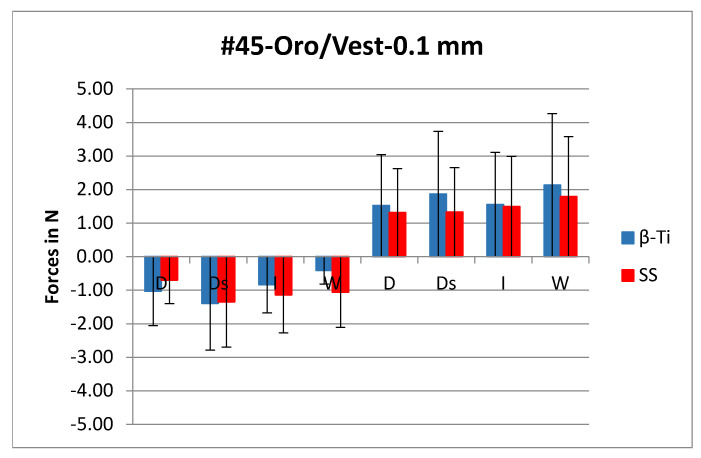
Graphic representation of the forces generated at the 0.1mm of the orovestibular movement of the premolar (#45). SS: stainless steel; β-Ti: beta titanium; D: discovery^®^ MIM; Ds: discovery^®^ smart; W: WiN, I: Incognito™; N: Newton.

In most cases, the forces generated by the bracket/β-Ti combination were slightly lower than those generated from the combination of the SS wire with the same bracket appliance (Figure 2). On the contrary, in 20 out of 96 cases, the β-Ti wires generated slightly higher forces as shown in Figure 3. All these cases represented orovestibular movements.

## 4. Discussion

The force systems of labial and fully customized lingual bracket systems at teeth of different types, inclinations and positions were investigated. Due to the rigid nature of the selected wire types, it was impossible to experiment on a malocclusion model; thus, copies of the setup model were used to represent the final stage of the alignment.

According to the results of this study, the null hypothesis must be rejected since differences were observed between the dissimilar bracket appliances and the different wire alloys.

The selected bracket appliances had a 0.018” slot size with horizontal “edgewise” orientation of the long slot walls apart from the lingual brackets, which had vertical “ribbonwise” orientation of the long slot walls, whereas the incisor and canine lingual brackets also had a vertical slot opening. The vertical orientation of the inserted wire in the lingual slots explains the higher force values generated from the fully customized lingual appliances during the intrusion and extrusion in comparison with the values generated from the labial appliances during activation on the same axis. In addition, forces generated from the lingual systems during the intrusion/extrusion were higher from those produced during the orovestibular activation of the wires due to the abovementioned reason. The use of lingual appliances can also result in higher force values because of the reduced free wire length and increased wire stiffness at the anterior region, which results from the morphology of the lingual bracket systems [10,11,32,33]. An example is the higher forces generated at the canine area during the orovestibular activation. For the lingual appliances, the 0.018” × 0.025” β-Ti archwires are indicated as finishing wires in cases where high torque expression is needed, while the SS wires are indicated for stabilization and anchorage during orthodontic or orthognathic therapy and for anchorage in combination with the Herbst device. Taking into consideration the specific indications of the wires mentioned above, the higher force levels could be beneficial in clinical practice and, especially, in cases where the orthodontist seeks methods to increase anchorage. The distances between the central incisor (#41) and the lateral incisor (#42) for the Discovery^®^ MIM appliance and the lingual appliances were the same. The lingual interbracket distances between the lateral incisor (#42) and the canine (#43) were half of the labial interbracket distances. Due to the larger slot widths, the Win interbracket distance between the first (#44) and second premolar (#45) was smaller, while the Incognito^TM^ brackets had the same interbracket distance as the Discovery^®^ smart appliance. The lingual interbracket distances between the second premolar (#45) and the first molar (#46) were smaller in comparison with labial interbracket distances. For the lingual bracket systems, the “ribbonwise” wire orientation results in smaller orovestibular forces in comparison with the labial appliances. The interbracket distances, in combination with the wire orientation in each case, resulted in similar orovestibular forces for the labial and lingual appliances at the lateral incisor and premolar areas.

The effects of orthodontic force on dental pulp and the apical foramen are of great importance. Histological and metabolic pulp changes have been observed in patients under orthodontic treatment [2,34]. Moreover, apical root resorption (ARR) has been associated with intrusion forces [35,36]. Risk factors, such us the force magnitude, the age of the patient, the tooth type, the treatment duration and the range of movement, should be taken into consideration [36,37,38]. Variances observed between activations on the same axis (i.e., between intrusion and extrusion or oral and vestibular activation) have multifactorial etiology. Firstly, the selected wires faced resistance from the slot walls or the ligature depending on the activation direction. Since the ligatures are far more elastic than the slot walls, a force, which results against the ligatures, can be lower. Secondly, slight differences could result from inconsistent ligation pressure and unalike wire adjustment.

Forces varied between the five activation repetitions with the same wire material. This observation confirms previous investigations, which showed that the use of different pieces of wire of the same alloy and dimensions could result in diverse force values [39,40]. Furthermore, the consecutive wire insertions and sensor adjustments could result in different contact status between the selected wires and bracket appliances, which is a typical measurement error.

Several statistically significant variances that were registered between the four appliances had no clinical significance. Differences of a few micrometers might be statistically significant but do not affect the overall treatment outcome.

Several authors reported that β-Ti wires have lower elastic modulus and, thus, generate lower forces at the same amount of deflection in comparison with the SS wires [13,20,41]. In this study, the differences between the two wire alloys were small. A possible explanation is the higher static and kinetic frictional resistance of the β-Ti alloy in comparison with the stainless-steel wires [42,43,44,45]. Previous investigations proved the adherence between the β-Ti alloy and the stainless-steel bracket surfaces, which result in higher friction forces [46]. The beta-titanium wires also present higher surface roughness than the stainless-steel ones; however, the correlation between wire surface roughness and friction is still a controversial subject [44,47,48]. The width and height of each wire piece were registered, and the dimensions of the β-Ti wires were found slightly larger than those of the SS wires. Furthermore, due to the larger dimensions of the beta-titanium wires, the slot play was smaller, and the consequent friction forces increased. These factors could have affected the resulting force values.

### 4.1. Sources of Error

Errors relative to the obtained values could arise from various aspects of the experimental procedure, such as model scanning and duplication, positioning of the brackets, the wires and the sensor and sensor accuracy and statistical error of repetition. Model scanning proved to be a reliable method for digitizing the classic stone casts [49,50]. Stone cast duplication is very common in dentistry, and the precision of silicone has been analyzed before [51,52,53]. Moreover, measurements on stone models with the use of calipers proved to be comparable with the use of three dimensional software [54]. The positioning of the brackets, the bonding procedure using transfer keys, the wire adjustments and ligation were performed by the same examiner following a standardized protocol. It is difficult to quantify this error source, which has an effect on the positioning accuracy of brackets and wires in the measurement model. However, as all the steps have been performed by one examiner using transfer keys and identical material was used, we assume that the effect on the overall error might be neglected compared to the other error sources.

The maximum sensor error in linearity is 0.3% and 1.8% due to cross-talk, resulting in an overall sensor error of 0.02 N for forces and 0.5 Nmm for torques [30], which is below 1% of the measured maximum forces. The positioning resolution of the OMSS is 1 μm, which again is less than 1 % of the maximum activation and could have similar effects on the force errors [30].

Finally, a possible additional source of error in the force measurements might be the wire/slot play of the wire inserted into the slot of the measurement bracket. Although the measurement bracket is adjusted to deliver force and torque readings of 0.0 N (Nmm) any time prior to start of the activation measurement, we cannot exclude the possibility that the wire slot play might have a varying influence on the individual force/torque measurements. Taking the nominal slot height of 0.457 mm (0.018”) and wire dimensions from Table 3, it becomes obvious that the wire/slot play might reach values of up to 0.037 mm for Incognito and 0.025 mm for the Win appliances. For the standard appliances, maximum play reaches 0.017 mm. Thus, wire/slot play might reach 10% or even more of the maximum measured deflection and obviously seems to have the highest influence on measured force error of these activation measurements.

In order to reduce random error, the force measurements were repeated five times in each direction, and only the mean values were compared. By calculating the overall error from the above cited error sources using Gaussian’s law of error propagation, we can estimate a maximum systematic and measurement error of 15% within which the wire/slot play has decisive influence. This is consistent with clinical experience that wire/slot play has decisive influences on tooth positions in the final adjustment phase.

### 4.2. Limitations

The limitations of this experimental investigation are as follows: (1) experimentation in an idealized environment (simulation device) without periodontal ligament, mobility of the adjacent teeth, occlusion, muscle forces and saliva; and (2) the use of stainless-steel ligatures. Stainless-steel ligatures result in reduced friction forces compared to elastic ligatures [55]. Previous investigations presented controversial results regarding the influence of saliva on friction and the resultant orthodontic forces [56,57]. In addition, the fully customized lingual appliances are always customized on a VTO setup model, while the labial appliances were not. The force values described above might differ from those generated in clinical practice because of these limitations; thus, the obtained values are used only as a standard of comparison between the dissimilar bracket systems and wire alloys.

## 5. Conclusions

The differences between the force values generated from the β-Ti and the SS 0.018” × 0.025” wires were small. Within the limits of this study, we could assume that possibly only one of the two archwire alloys could be used as a part of a wire sequence during the orthodontic therapy; however, further investigation is needed. Specifically, the moment values generated from the particular bracket/wire combinations should also be investigated in order to draw conclusions. Higher forces (5–53%) were generated in some cases from the lingual appliances in comparison with the forces produced from the labial appliances when tested with the same wire. These forces could be beneficial in clinical practice in cases where increased anchorage is needed. 

## Figures and Tables

**Table 1:** Physical, mechanical and thermal properties of archwires. The wire dimensions in millimeters were calculated from manufacturers’ values (0.018” × 0.025” and 0.0182” × 0.025”). Young’s moduli were calculated from Proffit [13].

		Dimensions (mm)	Young’s Modulus (GPa)	Temperature Dependence
β-Ti	Incognito^TM^	0.635 × 0.462	72	None
	WiN	0.635 × 0.457		
	Dentaurum	0.457 × 0.635		
SS	Incognito^TM^	0.635 × 0.457	200	None
	WiN	0.635 × 0.457		
	Dentaurum	0.457 × 0.635		

**Table 2:** Slot lengths (according to ISO27020:2019) and distance between the adjacent slots of all the appliances used in this study.

	**Slot Length (mm)**			
	**Discovery^®^ MIM**	**Discovery^®^ Smart**	**WiN**	**Incognito™**
Lower left 1st bicuspid (#34)	3.3	2.7	3.1	2.5
Lower left cuspid (#33)	2.9	2.7	2.5	2.3
Lower left lateral incisor (#32)	2.6	2.3	2.1	2.4
Lower right central incisor (#41)	2.6	2.3	2.1	2.4
Lower right lateral incisor (#42)	2.6	2.3	2.1	2.4
Lower right cuspid (#43)	3.0	2.7	2.5	2.4
Lower right 1st bicuspid (#44)	3.3	2.8	3.0	2.4
Lower right 2nd bicuspid (#45)	3.3	2.7	3.1	2.4
Lower right 1st molar (#46)	3.2	3.2	3.1	3.9
	**Distance Between Adjacent Slots (mm)**			
	**Discovery^®^ MIM**	**Discovery^®^ Smart**	**WiN**	**Incognito™**
#34–#33	4.5	4.9	3.0	3.4
#33–#32	4.2	4.9	2.8	3.0
#41–#42	3.1	3.7	3.0	2.9
#42–#43	4.6	5.3	2.2	2.2
#44–#45	3.6	4.0	3.3	4.1
#45–#46	5.7	6.6	4.7	4.4

**Table 3:** Measured dimensions of the selected wires. The nominal dimensions are 0.635 × 0.462 mm for beta-titanium wires received from Incognito^TM^ and 0.635 × 0.457 mm for the rest of the wires.

	Wire Dimensions (mm)			
	Discovery^®^ MIM	Discovery^®^ Smart	WiN	Incognito™
SS1	0.440 × 0.624	0.442 × 0.626	0.632 × 0.457	0.622 × 0.451
SS2	0.441 × 0.624	0.440 × 0.622		0.623 × 0.450
SS3	0.441 × 0.622	0.444 × 0.620		0.623 × 0.453
SS4	0.445 × 0.623	0.443 × 0.621		0.620 × 0.452
SS5	0.445 × 0.622	0.440 × 0.622		0.620 × 0.452
β-Ti1	0.450 × 0.631	0.450 × 0.633	0.635 × 0.445	0.623 × 0.455
β-Ti2	0.443 × 0.628	0.450 × 0.628		0.623 × 0.455
β-Ti3	0.446 × 0.632	0.449 × 0.632		0.623 × 0.454
β-Ti4	0.449 × 0.630	0.450 × 0.632		0.624 × 0.455
β-Ti5	0.446 × 0.631	0.450 × 0.630		0.620 × 0.456

SS: stainless steel; β-Ti: beta titanium.

**Table 4:** Intrusion/extrusion and orovestibular force values generated during positive activation at a specific tooth (canine, lateral incisor and premolar) area using the four different bracket appliances combined with the stainless-steel wire at 0.1 mm and 0.2 mm.

Act+		Teeth under Examination											
		#33				#42				#45			
		0.1 mm		0.2 mm		0.1 mm		0.2 mm		0.1 mm		0.2 mm	
Brackets	Wires	Fx	Fz	Fx	Fz	Fx	Fz	Fx	Fz	Fx	Fz	Fx	Fz
		(N)	(N)	(N)	(N)	(N)	(N)	(N)	(N)	(N)	(N)	(N)	(N)
D	SS	−1.3 ^a^	−1.1 ^r^	−2.7 ^c^	−1.8 ^u^	−1.7 ^f^	−1.5 ^x^	−3.4 ^i,k^	−2.2 ^j^	−1.9 ^m,n^	−0.7 ^φ^	−3.3 ^p^	−1.5 ^ω^
Ds	SS	−2.0 ^b^	−1.0 ^r^	−3.5 ^d^	−1.6 ^u^	−1.3 ^g^	−1.0 ^y^	−3.0 ^k^	−1.9 ^l^	−1.5 ^m^	−1.3 ^ψ^	−2.7 ^p^	−2.2 ^β^
W	SS	−2.5 ^b^	−0.6 ^s^	−3.0 ^c,d^	−1.0 ^v^	−2.1 ^f^	−0.9 ^y^	−2.8 ^k^	−1.4 ^θ^	−2.9 ^n^	−1.1 ^ψ^	−4.7 ^q^	−1.7 ^ω,β^
I	SS	−2.5 ^b^	−1.7 ^t^	−4.2 ^e^	−2.8 ^w^	−2.6 ^h^	−1.7 ^z^	−3.9 ^i^	−2.8 ^λ^	−2.3 ^o^	−1.2 ^ψ^	−4.7 ^q^	−1.9 ^ω,β^
*p* value for ANOVA		0.000	0.000	0.000	0.000	0.000	0.000	0.004	0.000	0.000	0.001	0.000	0.013

Act+: Positive activation; Fx: Forces generated during the intrusion; Fz: Forces generated during the vestibular movement (see Figure I). D: discovery^®^ MIM; Ds: discovery^®^ smart; W: WiN; I: Incognito™; N: Newton. ^a–z^: Values marked with the same letter do not differ according to Student–Newman–Keuls post-hoc tests.

**Table 5:** Intrusion/extrusion and orovestibular force values generated during negative activation at a specific tooth (canine, lateral incisor and premolar) area using the four different bracket appliances combined with the stainless-steel wire at 0.1 mm and 0.2 mm.

Act-		Teeth under Examination											
		#33				#42				#45			
		0.1 mm		0.2 mm		0.1 mm		0.2 mm		0.1 mm		0.2 mm	
Brackets	Wires	Fx	Fz	Fx	Fz	Fx	Fz	Fx	Fz	Fx	Fz	Fx	Fz
		(N)	(N)	(N)	(N)	(N)	(N)	(N)	(N)	(N)	(N)	(N)	(N)
D	SS	1.7 ^a^	1.6 ^q^	2.3 ^c^	2.5 ^t^	2.4 ^f^	2.0 ^w^	3.2 ^i^	2.8 ^x^	1.8 ^l^	1.3 ^y^	2.5 ^o^	2.1 ^θ^
Ds	SS	1.7 ^a^	1.3 ^q^	2.3 ^c^	1.9 ^u^	1.4 ^g^	2.2 ^w^	2.2 ^j^	3.0 ^x^	1.8 ^l^	1.3 ^y^	2.4 ^o^	2.3 ^θ^
W	SS	3.1 ^b^	3.1 ^r^	3.9 ^d^	3.4 ^v^	3.0 ^h^	2.2 ^w^	4.1 ^k^	2.8 ^x^	3.0 ^m^	1.8 ^z^	3.6 ^p^	2.2 ^θ^
I	SS	3.0 ^b^	2.0 ^s^	4.6 ^e^	2.7 ^t^	2.8 ^h^	2.4 ^w^	3.9 ^k^	3.0 ^x^	2.4 ^n^	1.5 ^y,z^	3.3 ^p^	2.4 ^θ^
*p* value for ANOVA		0.000	0.000	0.000	0.000	0.000	0.699	0.000	0.451	0.000	0.000	0.022	0.388

Act-: Negative activation; Fx: Forces generated during the extrusion; Fz: Forces generated during the oral movement. D: discovery^®^ MIM; Ds: discovery^®^ smart; W: WiN; I: Incognito™; N: Newton. ^a–z^: Values marked with the same letter do not differ according to Student–Newman–Keuls post-hoc tests.

**Table 6:** Intrusion/extrusion and orovestibular force values generated during positive activation at a specific tooth (canine, lateral incisor and premolar) area using the four different bracket appliances combined with the beta-titanium wire at 0.1 mm and 0.2 mm.

Act+		Teeth under Examination											
		#33				#42				#45			
		0.1 mm		0.2 mm		0.1 mm		0.2 mm		0.1 mm		0.2 mm	
Brackets	Wires	Fx	Fz	Fx	Fz	Fx	Fz	Fx	Fz	Fx	Fz	Fx	Fz
		(N)	(N)	(N)	(N)	(N)	(N)	(N)	(N)	(N)	(N)	(N)	(N)
D	β-Ti	−1.1 ^b^	−1.0 ^s^	−2.1 ^d^	−1.5 ^u^	−1.4 ^f^	−1.2 ^x^	−2.7 ^i^	−2.0 ^y^	−1.4 ^k^	−1.0 ^θ^	−2.6 ^o^	−1.5 ^ψ^
Ds	β-Ti	−1.8 ^a^	−0.9 ^s^	−3.4 ^e^	−1.5 ^u^	−1.1 ^f^	−1.2 ^x^	−2.6 ^i^	−1.9 ^y^	−1.2 ^l^	−1.4 ^λ^	−2.3 ^p^	−2.1 ^ω^
W	β-Ti	−1.9 ^a^	−1.7 ^t^	−2.3 ^d^	−2.8 ^v^	−2.2 ^g^	−1.1 ^x^	−3.0 ^i^	−1.8 ^y^	−2.1 ^m^	−0.4 ^φ^	−4.0 ^q^	−0.7 ^γ^
I	β-Ti	−1.4 ^c^	−1.4 ^t^	−2.5 ^d^	−2.2 ^w^	−2.6 ^h^	−1.4 ^x^	−4.1 ^j^	−2.5 ^z^	−1.9 ^n^	−0.8 ^θ^	−3.8 ^r^	−1.4 ^ψ^
*p* value for ANOVA		0.000	0.000	0.000	0.000	0.000	0.264	0.001	0.000	0.000	0.001	0.000	0.013

Act+: Positive activation; Fx: Forces generated during the intrusion; Fz: Forces generated during the vestibular movement. D: discovery^®^ MIM; Ds: discovery^®^ smart; W: WiN; I: Incognito™; N: Newton. ^a–z^: Values marked with the same letter do not differ according to Student–Newman–Keuls post -hoc tests.

**Table 7:** Intrusion/extrusion and orovestibular force values generated during negative activation at a specific tooth (canine, lateral incisor and premolar) area using the four different bracket appliances combined with the beta-titanium wire at 0.1 mm and 0.2 mm.

Act-		Teeth under Examination											
		#33				#42				#45			
		0.1 mm		0.2 mm		0.1 mm		0.2 mm		0.1 mm		0.2 mm	
Brackets	Wires	Fx	Fz	Fx	Fz	Fx	Fz	Fx	Fz	Fx	Fz	Fx	Fz
		(N)	(N)	(N)	(N)	(N)	(N)	(N)	(N)	(N)	(N)	(N)	(N)
D	β-Ti	1.5 ^a^	1.4 ^q^	2.1 ^c^	2.2 ^t^	1.9 ^e^	1.8 ^w^	2.7 ^h^	2.5 ^y,z^	1.5 ^k^	1.5 ^θ^	2.1 ^n^	2.4 ^λ^
Ds	β-Ti	1.6 ^a^	1.3 ^q^	2.2 ^c^	1.9 ^t^	1.4 ^f^	1.9 ^w,x^	2.2 ^i^	2.7 ^y^	1.7 ^k^	1.9 ^θ,ω^	2.3 ^n^	2.8 ^λ^
W	β-Ti	2.6 ^b^	2.6 ^r^	3.4 ^d^	3.5 ^u^	2.6 ^g^	1.6 ^w,x^	3.4 ^j^	2.9 ^z^	2.7 ^l^	2.1 ^ω^	3.2 ^o^	2.5 ^λ^
I	β-Ti	2.5 ^b^	2.1 ^s^	3.1 ^d^	2.7 ^v^	2.5 ^g^	2.2 ^x^	3.3 ^j^	2.2 ^y^	2.1 ^m^	1.6 ^θ^	2.8 ^p^	2.0 ^ψ^
*p* value for ANOVA		0.000	0.000	0.000	0.000	0.000	0.054	0.000	0.006	0.000	0.004	0.000	0.002

Act-: Negative activation; Fx: Forces generated during the extrusion; Fz: Forces generated during the oral movement. D: discovery^®^ MIM; Ds: discovery^®^ smart; W: WiN; I: Incognito™; N: Newton. ^a–z^: Values marked with the same letter do not differ according to Student–Newman–Keuls post-hoc tests.

## Data Availability

The data presented in this study are available on request from the corresponding author. The data are not publicly available due to the amount and file size of raw data.
